# Supplementary material for: Impact of Functional Polymorphisms on Drug Survival of Biological Therapies in Patients with Moderate-to-Severe Psoriasis
Source: Int J Mol Sci. 2023 May 12;24(10):8703. doi: 10.3390/ijms24108703 (PMC10218224; doi:10.3390/ijms24108703)
Supplement: Supplementary file 1 [file ijms-24-08703-s001.zip › ijms-2344143-supplementary figures.pdf]

International Journal of
Molecular Sciences

Supplementary Materials Figures

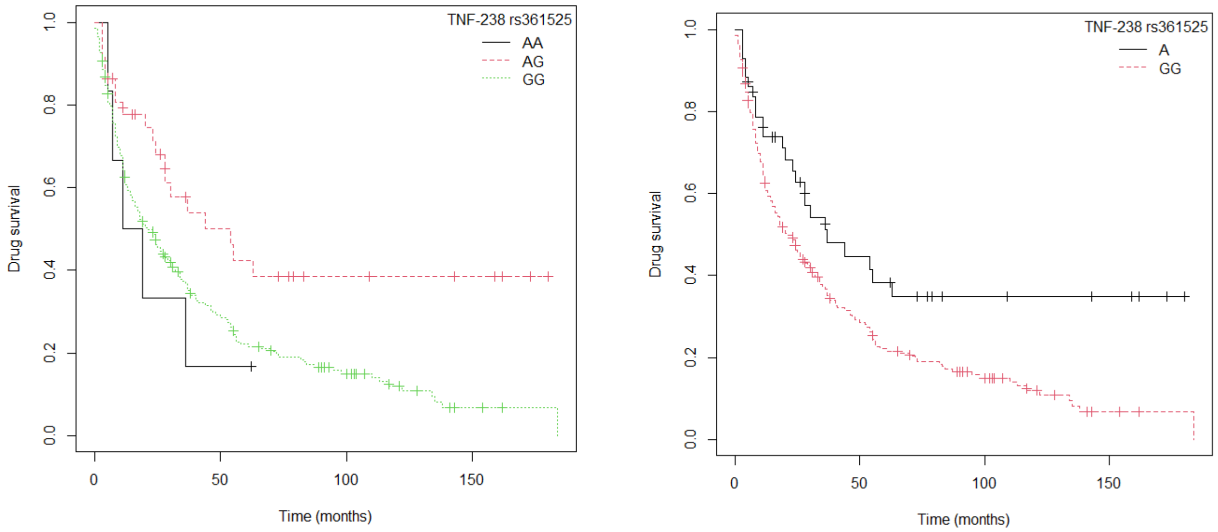

(**a**) (**b**)

Figure S1. The Kaplan-Meier survival curves of anti-TNF and *TNF*-238 rs361525. (**a**) genotypes; (**b**) Allele A.

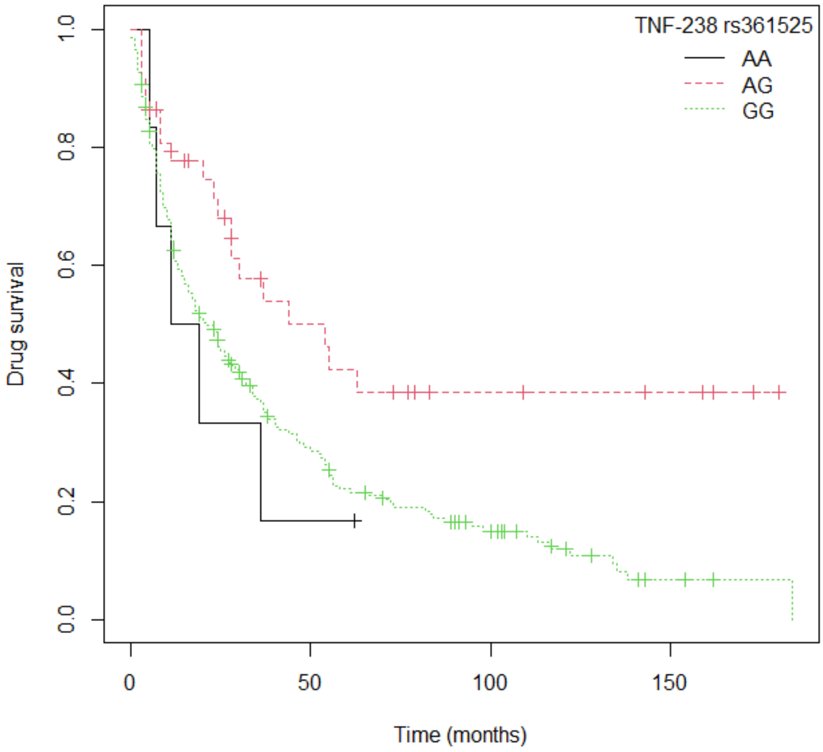

Figure S2. The Kaplan-Meier survival curves of anti-TNF and *TNF*-308 rs1800629, genotypes.

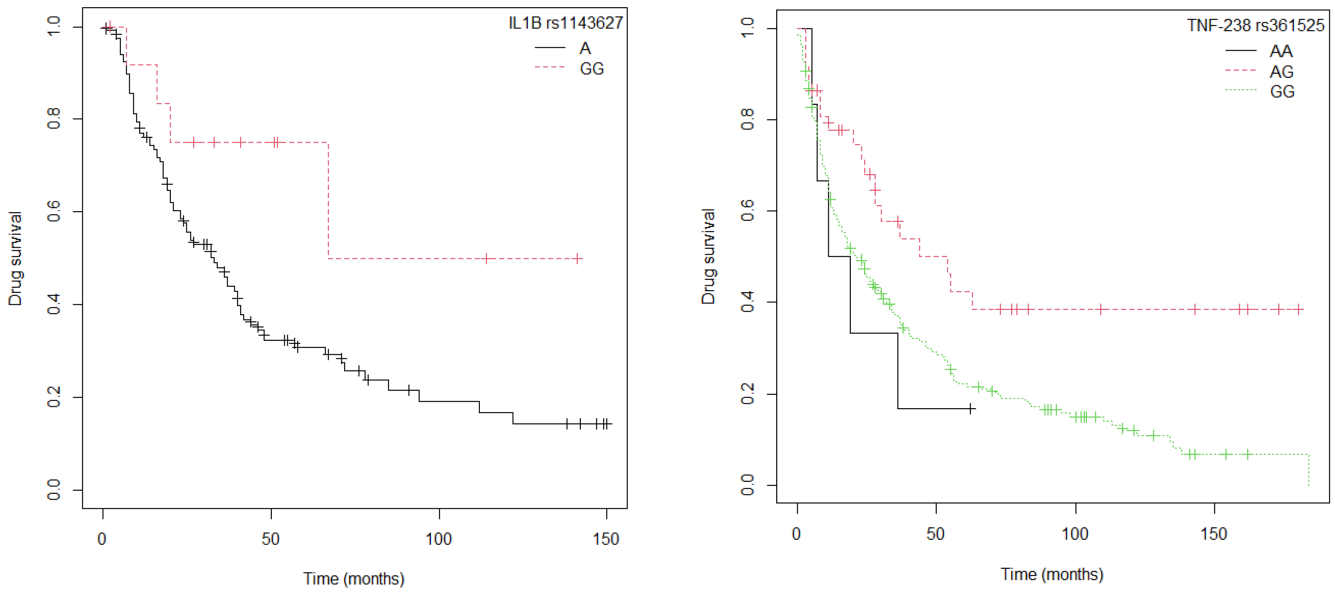

(**a**) (**b**)

Figure S3. The Kaplan-Meier survival curves of UTK and *IL1B* rs1143627. (**a**) genotypes, (**b**) allele A vs. GG.

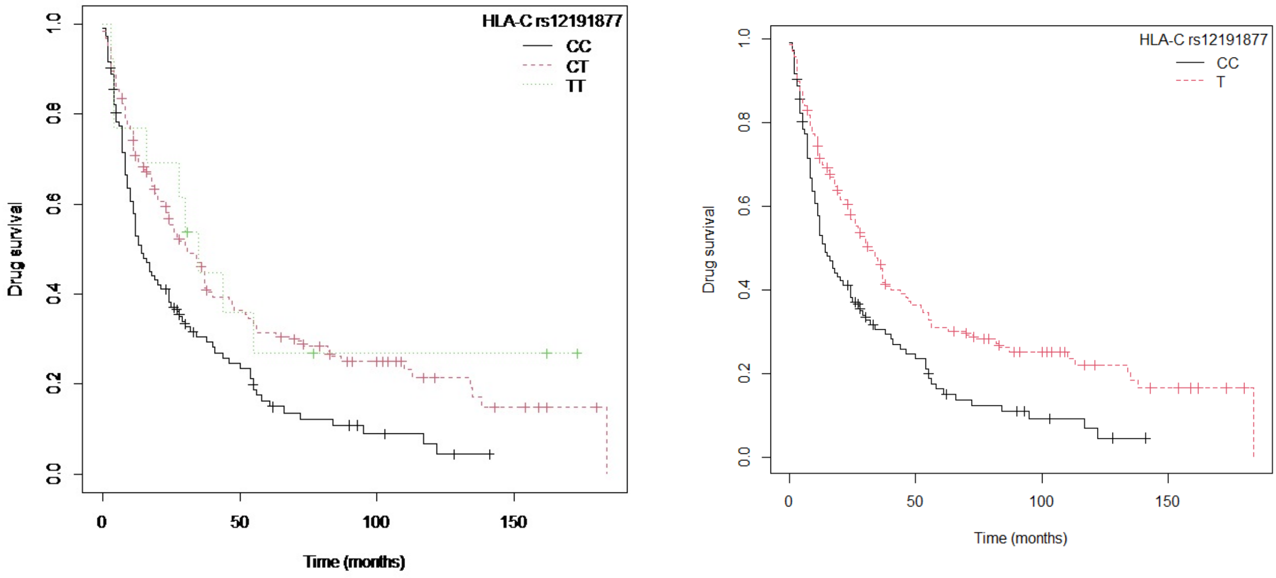

(**a**) (**b**)

Figure S4. The Kaplan-Meier survival curve of UTK and *HLA-C* rs12191877. (**a**) Allele T, (**b**) genotypes.
