# Supplementary material for: Impact of Functional Polymorphisms on Drug Survival of Biological Therapies in Patients with Moderate-to-Severe Psoriasis
Source: Int J Mol Sci. 2023 May 12;24(10):8703. doi: 10.3390/ijms24108703 (PMC10218224; doi:10.3390/ijms24108703)
Supplement: Supplementary file 1 [file ijms-24-08703-s001.zip › Table S1. Patients Description.pdf]

Table S1. Patients' description diagnosis with moderate-to-severe psoriasis treated with Anti-TNF and Anti-IL12/23.

**Variable**	**Baseline (N=198)**		
	**N**	**%**	**Mean ± standard deviation**
**Sex**			
Female	104	52.53	-
Male	94	47.47	-
**Age at baseline**	173	-	52.81 ± 14.17
<52 years	72	41.62	-
>52 years	101	58.38	-
**BMI at baseline**	196	-	27.09 (23.99-31.23)
Normal weight	68	35.42	-
Overweight	64	33.33	-
Obesity Type I	35	18.23	-
Obesity Type II	15	7.81	-
Obesity Type III	10	5.21	-
**COMORBIDITIES**			
**Psoriatic Arthritis**	89	44.95	-
**Hypertension**	65	32.83	-
**Dyslipidemia**	78	39.39	-
**Other comorbidities**	129	65.15	
**Age diagnosis PS**	198	-	28 (18-42)
**Family history PS**	115	58.38	-
**Type of PS**			
Plaque	87	43.94	-
Pustular	4	2.02	-
Inverse	2	1.01	-
Guttate	6	3.03	-
Combination 2 types of PS	73	36.87	-
Combination 3 types of PS	25	12.63	-
Combination 4 types of PS	1	0.51	-
**Location of lesions PS**			
Trunk and lower and upper limbs	178	89.9	-
Scalp and face	127	64.14	-
Nails	82	41.41	-
Palmoplantar	28	14.14	-
Flexures	55	27.78	-
Genital	23	11.79	-

PS: Psoriasis.

Qualitative variables are shown as numbers (percentage, %). Lilliefors (Kolmogorov-Smirnov) normality test. Quantitative variables with a normal distribution are shown as mean ± standard deviation. Quantitative variables with a non-normal distribution are shown as p_{50} (p_{25} - p_{75}).
