# Supplementary material for: Impact of Functional Polymorphisms on Drug Survival of Biological Therapies in Patients with Moderate-to-Severe Psoriasis
Source: Int J Mol Sci. 2023 May 12;24(10):8703. doi: 10.3390/ijms24108703 (PMC10218224; doi:10.3390/ijms24108703)
Supplement: Supplementary file 1 [file ijms-24-08703-s001.zip › Table S10. SNP- Drug survival- UTK .pdf]

Table S10. Polymorphisms and association with drug survival of the Anti-IL12/23 treatment patients**.**

Gene	SNPs	Genotype	Drug Survival (months)- Anti-IL12/23- (N=132)							
			N	Events	MST (mo)	IC95%	Log-Rank p-value	Univariate Cox Model HR	Univariate Cox Model IC95%	Univariate Cox Model p-value
HLA-B/ MICA	*rs13437088*	AA	13	8	30	9-NA	0.5			
		AC	58	36	33	25-57				
		CC	60	42	36	21-67				
		A	71	44	33	23-57	0.4			
		C	118	78	36	25-44	0.5			
HLA-C	*rs12191877*	CC	70	51	25	20-42	0.2			
		CT	57	34	39	32-85				
		TT	5	2	NA	25-NA				
		C	127	85	36	25-42	0.4			
		T	62	36	39	32-112	**0.06**	0.668	0.43-1.02	**0.066**
TNF-α	*(TNF-238) rs361525*	GG	105	69	37	23-48	0.4			
		AG	26	18	33	21-NA				
		AA	1	0	NA	NA-NA				
		G	131	87	36	25-42	0.2			
		A	27	18	34.5	21-NA	1			
	(TNF-857) rs1799724	CC	101	68	34	21-48	0.6			
		CT	31	19	36	25-NA				
		T	31	19	36	25-NA	0.6			
	(TNF-308) rs1800629	GG	91	60	36	25-46	0.7			
		AG	41	27	32	23-94				
		A	41	27	32	23-94	0.7			
	(TNF-1031) rs1799964	TT	76	52	34	20-48	0.6			
		CT	42	27	36	27-NA				
		CC	14	8	40	19-NA				
		T	118	79	34	25-44	0.4			
		C	56	35	36	27-71	0.4			
TNFRSF1B	*rs1061622*	TT	77	53	36	20-48	0.6			
		GT	45	28	37	23-67				
		GG	10	6	26	21-NA				
		T	122	81	36	25-44	0.6			
		G	55	34	37	23-67	0.4			
TNFAIP3	*rs610604*	GG	24	19	33	19-67	0.5			
		GT	62	39	36	23-57				
		TT	46	29	40	21-94				
		G	86	58	33	23-42	0.6			
		T	108	68	37	26-57	0.2			
IL1B	*rs1143623*	CC	75	48	40	24-75	0.2			
		CG	49	36	27	20-40				
		GG	8	3	67	16-NA				
		C	124	84	34	25-41	0.2			
		G	57	39	34	20-66	0.3			
	rs1143627	GG	13	4	67	67-NA	0.03	0.293	0.10-0.81	0.019
		AG	62	45	27	18-40		1		
		AA	57	38	40	23-78		0.701	0.45-1.08	0.111
		G	75	49	34	23-48	0.4			
		A	119	83	33	24-41	0.03	0.352	0.12-0.96	0.0417
IL6	*rs1800795*	CC	19	14	42	33-NA	0.6			
		CG	50	34	32	19-78				

		GG	63	39	37	23-57				
		C	69	48	34	21-67	0.7			
		G	113	73	32	23-44	0.5			
IL12β	*rs3213094*	CC	71	45	34	23-71	0.8			
		CT	56	39	36	23-57				
		TT	4	2	32	9-NA				
		C	127	84	36	25-44	0.5			
		T	60	41	36	25-57	0.9			
	rs2546890	AA	22	13	41	23-NA	0.6			
		AG	75	49	37	24-48				
		GG	35	25	33	16-67				
		A	97	62	37	25-48	0.3			
		G	110	74	36	24-44	0.5			
TIRAP	*rs8177374*	CC	88	58	37	26-48	1			
		CT	33	22	26	21-NA				
		TT	11	7	40	20-NA				
		C	121	80	34	25-44	1			
		T	44	29	27	21-NA	0.8			
PGLYR4-24	*rs2916205*	CT	34	24	37	16-122	0.9			
		TT	98	63	34	25-48				
		C	34	24	37	16-122	0.9			
CDKAL1	*rs6908425*	TT	3	3	32	26-NA	0.9			
		CT	33	20	41	20-NA				
		CC	96	64	34	24-46				
		T	36	23	40	23-85	0.7			
		C	129	84	36	25-44	0.8			
CD84	***rs6427528***	AA	1	1	10	NA-NA	**0.07**	1		
		AG	38	26	23	18-40		0.261	0.03-1.97	0.193
		GG	93	60	40	27-66		0.181	0.02-1.34	**0.095**
		A	39	27	23	18-40	**0.09**	0.676	0.42-1.06	**0.093**
		G	131	86	36	26-44	**0.08**	0.202	0.03-1.49	0.118
IL17RA	*rs4819554*	GG	5	2	57	57-NA	0.4			
		AG	36	23	21	15-NA				
		AA	91	62	36	26-44				
		G	41	25	27	18-NA	0.9			
		A	127	85	34	25-41	0.2			
IL23R	*rs11209026*	GG	118	76	39	26-48	0.2			
		AG	14	11	18.5	12-NA				
		A	14	11	18.5	12-NA	0.2			
TLR2	*rs4696480*	TT	23	15	33	21-NA	0.8			
		AT	56	41	32	21-44				
		AA	49	29	39	18-NA				
		T	79	56	32	23-41	0.7			
		A	105	70	34	23-48	0.7			
	rs11938228	CC	63	39	36	19-67	0.5			
		AC	48	35	32	20-48				
		AA	18	12	40	23-NA				
		C	111	74	32	23-48	0.4			
		A	66	47	33	23-42	0.7			
TLR5	*rs5744174*	AA	45	34	25	18-40	0.1			
		AG	66	43	36	23-71				
		GG	21	10	41	37-NA				
		A	111	77	32	23-44	0.2			
		G	87	53	41	26-67	**0.06**	0.665	0.43-1.03	**0.066**
TLR9	*rs352139*	TT	32	21	41	25-72	0.5			

		CT	70	48	26	19-40				
		CC	29	17	40	25-NA				
		T	102	69	32	23-41	0.4			
		C	99	65	32	23-42	0.6			
PDE3A SLCO1C1	*rs11045392 rs3794271*	TT	15	8	48	23-NA	0.04	0.442	0.21-0.94	0.036
		CT	57	36	37	26-94		0.649	0.41-1.02	**0.06**
		CC	59	43	25	18-40		1		
		T	72	44	40	32-78	0.02	0.600	0.39-0.92	0.0196
		C	116	79	33	24-41	0.1			
FCGR2A	*rs1801274*	AA	54	39	33	21-48	0.6			
		AG	53	33	36	21-78				
		GG	24	15	37	18-NA				
		A	107	72	33	24-44	0.5			
		G	77	48	37	25-72	0.3			
FCGR3A	*rs396991*	AA	47	34	23	18-48	0.3			
		AC	63	40	39	27-67				
		CC	21	12	57	20-NA				
		A	110	74	34	24-41	0.5			
		C	84	52	40	32-67	0.1			

MST: median survival time (months); HR: hazard ratio; IC95%: 95% confidence interval; NA: not achieved; Anti-IL12/23: interleukin 12 and interleukin 23 inhibitor (ustekinumab); PS: Psoriasis.

Statistically significant values are colored in grey, with a tendency to significance in bold.
