# Supplementary material for: Impact of Functional Polymorphisms on Drug Survival of Biological Therapies in Patients with Moderate-to-Severe Psoriasis
Source: Int J Mol Sci. 2023 May 12;24(10):8703. doi: 10.3390/ijms24108703 (PMC10218224; doi:10.3390/ijms24108703)

**Table S2. Drug survival for all biological therapies according to therapeutic targets.-DUDA**

| Drugs by therapeutic target | Drug Survival- All treatments | | | | | | | |
|---|---|---|---|---|---|---|---|---|
| | N | Events | MST (mo) | IC95% | Log-Rank p-value | Univariate Cox Model | | |
| | | | | | | HR | IC95% | p-value |
| **ANTI-TNF** | 247 | 193 | 24 | 18-31 | **0.07** | 1 | | |
| **ANTI-IL12/23** | 132 | 87 | 36 | 25-44 | | 0.75 | 0.58-0.97 | 0.0316 |

MST: median survival time (months)
HR: hazard ratio
IC95%: 95% confidence interval
NA: not achieved
Anti-TNF: Tumour Necrosis Factor inhibitor (Adalimumab, Certolizumab pegol, Etanercept and Infliximab)
Anti-IL12/23: interleukin 12 and interleukin 23 inhibitor (Ustekinumab)

Statistically significant values are coloured in grey, tendency to significance in bold.

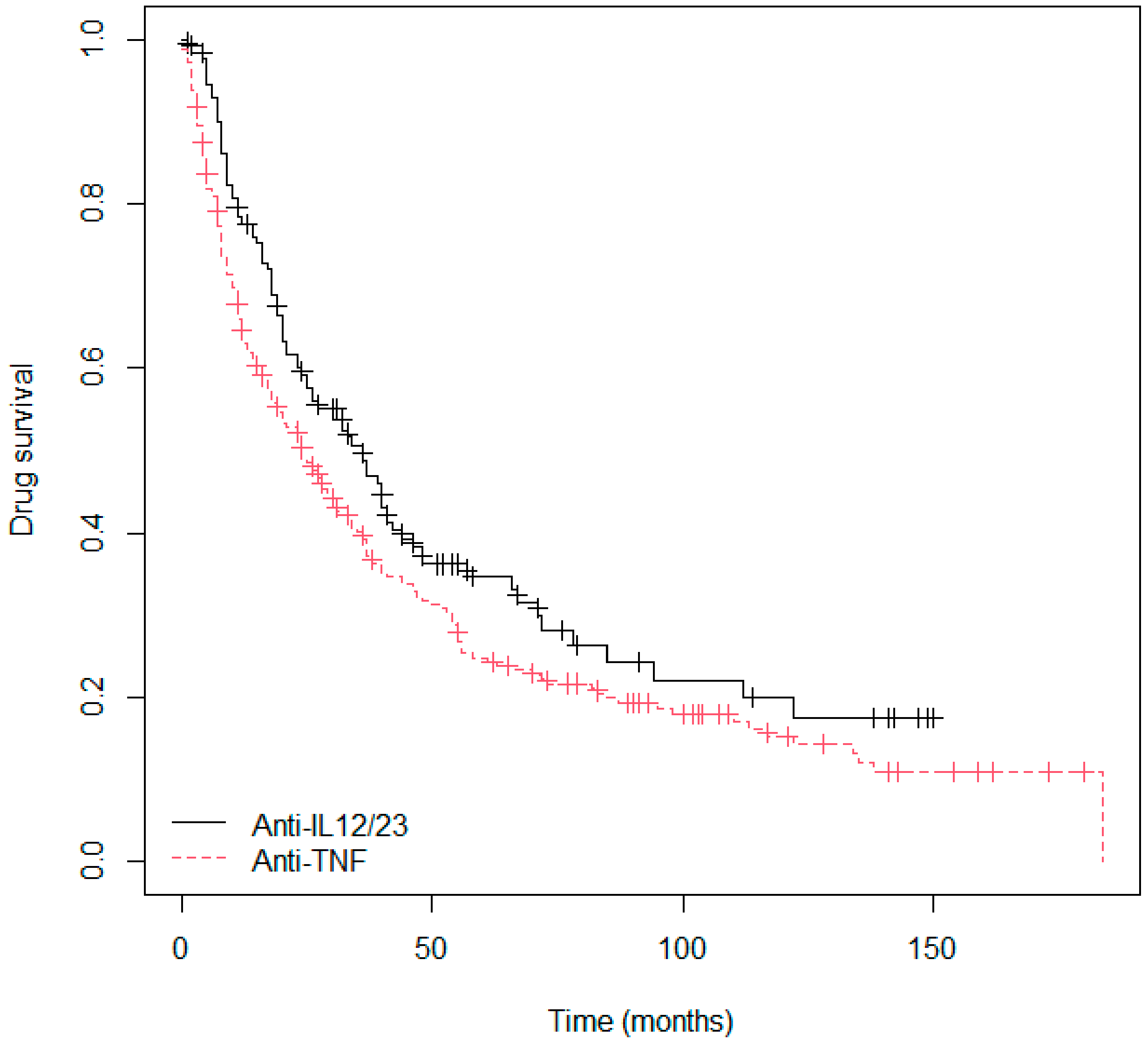

Supplement: Supplementary file 1 [file ijms-24-08703-s001.zip › Table S2. Drug Survival-Biological Therapy PS!.pdf]
