# Supplementary material for: Impact of Functional Polymorphisms on Drug Survival of Biological Therapies in Patients with Moderate-to-Severe Psoriasis
Source: Int J Mol Sci. 2023 May 12;24(10):8703. doi: 10.3390/ijms24108703 (PMC10218224; doi:10.3390/ijms24108703)
Supplement: Supplementary file 1 [file ijms-24-08703-s001.zip › Table S3. Discontinuation AntiTNF and Anti-IL12_23.pdf]

Table S3. Observed number and causes of discontinuation among the different drug exposure groups during the follow-up time.

Table S3A. Percentages are done on the total drug discontinuations.

**Reason for drug discontinuation**	**Anti-TNF N (%)**	**Anti-IL12/23 N (%)**	**Total**
Lack of efficacy	121 (62.7)	67 (77.0)	188
Adverse event	42 (21.8)	13 (14.9)	55
Remission	15 (7.8)	2 (2.3)	17
Other reasons	15 (7.8)	5 (5.7)	20
Total discontinuations	193	87	280

Anti -TNF: Tumour Necrosis Factor inhibitor (adalimumab, certolizumab pegol, etanercept and infliximab)
Anti-IL12/23: interleukin 12 and interleukin 23 inhibitor (ustekinumab)

Table S3B. Statistical analysis of the correlation between the group of drugs studied and the reason for discontinuation using the X^2 or Fisher test.

	**Anti-TNF (yes/no)**			**Anti-IL12/23 (yes/no)**		
	**$CI_{95\%}$**	**OR**	**p-value**	**$CI_{95\%}$**	**OR**	**p-value**
Lack of efficacy (yes/no)	0.36-0.99	0.604	0.036	1.05-3.52	1.895	0.024
Adverse event (yes/no)	0.94-3.39	1.768	**0.056**	-	-	0.343
Remission (yes/no)	1.05-21.07	3.835	0.027	-	-	0.173*
Other reasons (yes/no)	-	-	0.354	-	-	0.216

*Fisher-test.

The results that are statistically significant ($p<0.05$) are coloured in grey, a tendency to significance in bold, and $CI_{95\%}$ and OR are also indicated.
