# Supplementary material for: Impact of Functional Polymorphisms on Drug Survival of Biological Therapies in Patients with Moderate-to-Severe Psoriasis
Source: Int J Mol Sci. 2023 May 12;24(10):8703. doi: 10.3390/ijms24108703 (PMC10218224; doi:10.3390/ijms24108703)
Supplement: Supplementary file 1 [file ijms-24-08703-s001.zip › Table S4. Charact Clin_ANTI-TNF.pdf]

Table S4. Clinical characteristics and association with drug survival of the 247 PS patients' ANTI-TNF.

**Characteristic**		**Drug Survival (months)- ANTI-TNF (N=247)**							
							**Univariate Cox Model**		
		**N**	**Events**	**MST (mo)**	**IC95%**	**Log-Rank p-value**	**HR**	**IC95%**	**p-value**
**Gender**	Female	122	97	19	12-28	0.1			
	Male	125	96	30	20-44				
**Age at baseline**		197	150	-	-	-	0.987	0.97-0.99	0.041
	<54 years	93	75	17	12-32	**0.09**	1		
	>54 years	104	75	24	15-38		0.754	0.54-1.04	**0.088**
**BMI at baseline**		245	192	-	-	-	1.029	1.00-1.01	0.023
	Normal weight	78	58	36	19-54	0.3			
	Overweight	90	72	24	18-37				
	Obesity type I	45	35	23	11-30				
	Obesity type II	17	13	9	7-NA				
	Obesity type III	11	10	12	11-NA				
**Comorbidities**									
	**Psoriatic Arthritis**	129	108	24	17-30	0.2			
	**Hypertension**	96	79	27	19-37	0.6			
	**Dyslipidemia**	111	88	29	20-38	0.6			
	**Other comorbidities**	160	126	23	16-30	0.1			
**Age diagnosis PS**		247	193	-	-	-	0.999	0.99-1.01	0.871
**Family history PS**		133	102	30	23-37	0.2			
**Type of PS**	Plaque	231	181	24	19-31	0.8			
	Other types PS	16	12	13	3-NA				
**Bio-naive**	Yes	138	101	31	24-46	0.007	1		
	No	109	92	15	11-26		1.473	1.10-1.95	0.007
**Treatment line general**		247	193	-	-	-	1.099	1.01-1.19	0.033
**Concomitant Treatment**	Acitretin	2	0	NA	NA-NA	0.02	$9.4*10^{-8}$	0-Inf	0.991
	Cyclosporine	4	4	9.5	5-NA		1.29	0.47-3.5	0.614
	Methotrexate	32	26	29	12-50		0.957	0.62-1.46	0.839
	Topics	55	34	37	24-110		0.611	0.41-0.89	**0.01**
	Monotherapy (No)	154	129	19	14-27		1		
**Adherent BT**	Yes	195	152	24	18-30	0.3			
	No	47	36	38	11-56				
**Baseline PASI**		132	93	-	-	-	1.011	0.99-1.03	0.351
	<10	68	45	29	11-53	0.4			
	>10	64	48	23	13-37				

MST: median survival time (months); HR: hazard ratio; IC95%: 95% confidence interval; NA: not achieved. Anti-TNF: Tumour Necrosis Factor inhibitor (adalimumab, certolizumab pegol, etanercept and infliximab); BMI: Body Mass Index; BT: Biological therapy; PASI: Psoriasis Area Severity Index; PS: Psoriasis.

Statistically significant values are colored in grey, with a tendency to significance in bold.
