# Supplementary material for: Impact of Functional Polymorphisms on Drug Survival of Biological Therapies in Patients with Moderate-to-Severe Psoriasis
Source: Int J Mol Sci. 2023 May 12;24(10):8703. doi: 10.3390/ijms24108703 (PMC10218224; doi:10.3390/ijms24108703)
Supplement: Supplementary file 1 [file ijms-24-08703-s001.zip › Table S5. Charact clinical-UTK-DEFdocx.pdf]

Table S5. Clinical characteristics and association with drug survival of the 132 psoriasis patients treated with Ustekinumab.

Characteristic		Drug Survival (months)- Anti-IL12/23 (N=132)							
		N	Events	MST (mo)	IC95%	Log-Rank p-value	Univariate Cox Model		
							HR	IC95%	p-value
**Gender**	Female	77	54	32	23-42	0.3			
	Male	55	33	39	23-94				
**Age at baseline**		127	84	-	-	-	1.01	0.99-1.02	0.203
**BMI at baseline**		132	87	-	-	-	1.02	0.99- 1.05	0.21
	Normal weight	36	21	37	27-NA	**0.08**	1		
	Overweight	42	29	25	20-46		1.419	0.81-2.50	0.223
	Obesity type I	31	21	33	21-78		1.253	0.68-2.3	0.466
	Obesity type II	12	11	14.5	8-NA		2.733	1.30-5.73	0.0078
	Obesity type III	7	4	48	18-NA		0.871	0.298-2.53	0.799
**Comorbidities**									
	**Psoriatic Arthritis** Yes	63	49	21	19-36	0.003	1.899	1.24-2.91	0.0033
	No	69	38	48	37-94		1		
	**Hypertension**	41	29	36	25-57	0.9			
	**Dyslipidemia**	57	39	25	20-42	0.2			
	**Other comorbidities**	94	60	36	25-48	0.4			
**Age diagnosis PS**		132	87	-	-	-	1.005	0.99-1.02	0.48
**Family history PS**		77	48	36	20-72	0.4			
**Type of PS**	Plaque	121	80	34	24-46	0.8			
	Other types of PS	11	7	37	15-NA				
**Bio-naive**		44	26	41	33-NA	**0.09**	0.671	0.42-1.06	**0.0887**
**Treatment line general**		132	87	-	-	-	1.006	0.90-1.12	0.911
**Concomitant Tt.**	Cyclosporine	2	2	11	8-NA	0.03	1		
	Methotrexate	20	15	37.5	25-NA		0.178	0.04-0.81	0.025
	Topics	47	28	41	32-122		0.153	0.04- 0.66	0.012
	Monotherapy (No)	63	42	23	18-44		0.224	0.05-0.95	0.043
**Adherent BT**	Yes	98	63	26	20-39	**0.08**	1		
	No	33	23	57	40-112		0.647	0.39-1.05	**0.0809**
**Baseline PASI**		107	69	-	-	-	0.998	0.95-1.05	0.938
	<8	55	38	37	21-67	0.5			
	>8	52	31	34	20-NA				

Concomitant Tt. : Concomitant Treatment; MST: median survival time (months); HR: hazard ratio; IC95%: 95% confidence interval; NA: not achieved. Anti-IL12/23: interleukin 12 and interleukin 23 inhibitor (ustekinumab); BMI: Body Mass Index; BT: Biological therapy; PASI: Psoriasis Area Severity Index; PS: Psoriasis.

Statistically significant values are colored in grey, with a tendency to significance in bold.
