# Supplementary material for: Impact of Functional Polymorphisms on Drug Survival of Biological Therapies in Patients with Moderate-to-Severe Psoriasis
Source: Int J Mol Sci. 2023 May 12;24(10):8703. doi: 10.3390/ijms24108703 (PMC10218224; doi:10.3390/ijms24108703)
Supplement: Supplementary file 1 [file ijms-24-08703-s001.zip › Table S6. Minor alleles frequencies of SNPs.pdf]

Table S6. Minor alleles frequencies of SNPs

Chr	SNP	Minor Allele	Major Allele	MAF
1	rs1061622	G	T	0.2447
1	rs2916205	C	T	0.1368
1	rs6427528	A	G	0.1526
1	rs11209026	A	G	0.05789
1	rs5744174	G	A	0.4158
1	rs1801274	G	A	0.4328
1	rs396991	C	A	0.4127
2	rs1143623	G	C	0.2263
2	rs1143627	G	A	0.3237
3	rs352139	T	C	0.4735
4	rs4696480	T	A	0.4286
4	rs11938228	A	C	0.3694
5	rs3213094	T	C	0.2132
5	rs2546890	A	G	0.4842
6	rs13437088	A	C	0.3439
6	rs12191877	T	C	0.3175
6	rs361525	A	G	0.1
6	rs1799724	T	C	0.1316
6	rs1800629	A	G	0.1263
6	rs1799964	C	T	0.2526
6	rs610604	G	T	0.3545
6	rs6908425	T	C	0.1684
7	rs1800795	C	G	0.3263
8	rs11465996	G	C	0.2683
11	rs8177374	T	C	0.2079
12	rs11045392	T	C	0.3162
22	rs4819554	G	A	0.2354

Chr: Cromosome; MAF: Minor Allele Frequency.
