# Supplementary material for: Impact of Functional Polymorphisms on Drug Survival of Biological Therapies in Patients with Moderate-to-Severe Psoriasis
Source: Int J Mol Sci. 2023 May 12;24(10):8703. doi: 10.3390/ijms24108703 (PMC10218224; doi:10.3390/ijms24108703)
Supplement: Supplementary file 1 [file ijms-24-08703-s001.zip › Table S7. Equilibrium Hardy-Weinberg Drug Survival.pdf]

Table S7. Equilibrium Hardy-Weinberg Drug Survival

Chr	SNP	Minor Allele	Major Allele	Genotype counts	Observed heterozygosity	Expected heterozygosity	p-value	p-value verified*
1	rs1061622	G	T	15/63/112	0.3316	0.3697	0.1689	-
1	rs2916205	C	T	2/48/140	0.2526	0.2362	0.5383	-
1	rs6427528	A	G	2/54/134	0.2842	0.2587	0.2613	-
1	rs11209026	A	G	1/20/169	0.1053	0.1091	0.4752	-
1	rs5744174	G	A	30/98/62	0.5158	0.4858	0.4564	-
1	rs1801274	G	A	39/83/64	0.4462	0.491	0.2323	-
1	rs396991	C	A	29/98/62	0.5185	0.4848	0.3715	-
2	rs1143623	G	C	10/66/114	0.3474	0.3502	0.8388	-
2	rs1143627	G	A	18/87/85	0.4579	0.4378	0.6199	-
3	rs352139	T	C	42/95/52	0.5026	0.4986	1	-
4	rs4696480	T	A	33/84/58	0.48	0.4898	0.8772	-
4	rs11938228	A	C	29/75/76	0.4167	0.4659	0.1529	-
5	rs3213094	T	C	9/63/118	0.3316	0.3354	0.8305	-
5	rs2546890	A	G	43/98/49	0.5158	0.4995	0.7713	-
6	rs13437088	A	C	23/84/82	0.4444	0.4513	0.872	-
6	rs12191877	T	C	11/98/80	0.5185	0.4334	0.007438	0.7377
6	rs361525	A	G	1/36/153	0.1895	0.18	0.6995	-
6	rs1799724	T	C	1/48/141	0.2526	0.2285	0.2091	-
6	rs1800629	A	G	0/48/142	0.2526	0.2207	0.04861	0.9694
6	rs1799964	C	T	11/74/105	0.3895	0.3776	0.8474	-
6	rs610604	G	T	24/86/79	0.455	0.4577	1	-
6	rs6908425	T	C	8/48/134	0.2526	0.2801	0.1922	-
7	rs1800795	C	G	21/82/87	0.4316	0.4397	0.8688	-
8	rs11465996	G	C	15/58/91	0.3537	0.3926	0.2314	-
11	rs8177374	T	C	11/57/122	0.3	0.3293	0.2675	-
12	rs11045392	T	C	20/77/88	0.4162	0.4324	0.6117	-
22	rs4819554	G	A	17/55/117	0.291	0.36	0.01382	0.9538

Chr: Cromosome; MAF: Minor Allele Frequency. P-value verified: Verification of linkage disequilibrium comparing with the frequencies described for the Iberian population. Statistically significant values are colored in grey.
