# Supplementary material for: Impact of Functional Polymorphisms on Drug Survival of Biological Therapies in Patients with Moderate-to-Severe Psoriasis
Source: Int J Mol Sci. 2023 May 12;24(10):8703. doi: 10.3390/ijms24108703 (PMC10218224; doi:10.3390/ijms24108703)
Supplement: Supplementary file 1 [file ijms-24-08703-s001.zip › Table S8. Desequilibrio de ligamiento.pdf]

Table S8. Desequilibrio de ligamiento

**Gene**	**Chr**	**BP**	**SNP**	**Chr**	**BP**	**SNP**	**R^2**	**D**
IL1B	2	112838252	rs1143623	2	112836810	rs1143627	0.595928	1
TLR2	4	153685974	rs4696480	4	153700794	rs11938228	0.526891	0.829
TNF	6	31575324	rs361525	6	31574531	rs1799964	0.295626	1
Chr: Chromosome; BP: Physical position (base-pair).								
