# Supplementary material for: Impact of Functional Polymorphisms on Drug Survival of Biological Therapies in Patients with Moderate-to-Severe Psoriasis
Source: Int J Mol Sci. 2023 May 12;24(10):8703. doi: 10.3390/ijms24108703 (PMC10218224; doi:10.3390/ijms24108703)
Supplement: Supplementary file 1 [file ijms-24-08703-s001.zip › Table S9. SNP_Drug survival_ ANTI-TNF.pdf]

Table S9. Polymorphisms and association with drug survival of the ANTI-TNF treatment patients.

OPCION 1: SNP PROTECTORES DEL RIESGO- 😊 RESPUESTA

Gene	**SNPs**	**Genotype**	**Drug Survival (months)- ANTI-TNF (N= 247)**							
			**N**	**Events**	**MST (mo)**	**IC95%**	**Log-Rank p-value**	**Univariate Cox Model**		
								**HR**	**IC95%**	**p-value**
HLA-B/ MICA	rs13437088	AA	40	30	34	24-55	0.7			
		AC	108	82	23	16-31				
		CC	98	80	20	12-35				
		A	148	112	25	19-34	0.4			
		C	206	162	23	16-30	0.7			
HLA-C	rs12191877	CC	107	90	14	11-24	0.003	1		
		CT	124	91	31	23-40		0.618	0.46-0.83	0.0014
		TT	13	9	35	16-NA		0.534	0.27-1.06	**0.0744**
		C	231	181	24	18-30	0.3			
		T	137	100	31	24-40	0.0007	0.609	0.46-0.81	0.00078
TNF	(*TNF-238)* rs361525	GG	204	169	21	16-29	0.009	0.815	0.33-1.99	0.6530
		AG	37	19	44	28-NA		0.401	0.15-1.08	**0.0702**
		AA	6	5	15	7-NA		1		
		G	241	188	24	19-31	0.5			
		A	43	24	37	24-NA	0.007	0.563	0.36-0.86	0.00871
	(*TNF-857)* rs1799724	CC	185	146	24	18-30	0.9			
		CT	60	45	28	16-52				
		TT	2	2	62	37-NA				
		C	245	191	24	18-30	0.7			
		T	62	47	28	16-52	0.8			
	(TNF-*308)* rs1800629	GG	184	137	26	18-37	0.04	1		
		AG	63	56	23	13-30		0.724	0.53-0.99	0.0434
		A	63	56	23	13-80	0.04	0.724	0.53-0.99	0.0434
	(TNF-1031) rs1799964	TT	145	124	20	15-27	0.003	0.947	0.55-1.62	0.8453
		CT	85	54	44	25-70		0.552	0.31-0.98	0.0438
		CC	17	15	20	11-NA		1		
		T	230	178	24	18-31	0.4			
		C	102	69	36	23-56	0.004	0.648	0.48- 0.87	0.00442
TNFRSF1B	rs1061622	TT	153	127	24	15-31	0.2			
		GT	70	48	29	23-54				
		GG	24	18	17	11-56				
		T	223	175	24	19-31	0.9			
		G	94	66	28	19-44	0.1			
TNFAIP3	rs610604	GG	24	18	15	8-NA	0.9			
		GT	124	96	24	17-37				
		TT	98	78	25	16-36				
		G	148	114	23	17-34	0.8			
		T	222	174	24	19-31	0.8			
IL1B	*rs1143623*	CC	146	114	23	17-31	0.6			
		CG	81	62	30	16-38				
		GG	20	17	15	8-46				
		C	227	176	24	19-31	0.4			
		G	101	79	28	14-37	0.6			
	rs1143627	GG	28	24	21.5	8-44	0.4			
		AG	105	77	34	20-41				
		AA	114	92	20	16-28				
		G	133	101	30	19-37	0.5			

		A	219	169	24	18-34	0.4			
IL6	*rs1800795*	CC	21	14	17	11-NA	0.7			
		CG	101	75	27	18-37				
		GG	125	104	23	16-34				
		C	122	89	25	18-37	0.4			
		G	226	179	24	19-34	0.8			
IL12β	*rs3213094*	CC	162	126	25	19-36	0.4			
		CT	78	60	24	13-37				
		TT	6	6	17.5	3-NA				
		C	240	186	24	19-31	0.2			
		T	84	66	23	13-32	0.5			
	rs2546890	AA	55	43	18	12-40	0.8			
		AG	138	108	23	17-34				
		GG	54	42	30	23-52				
		A	193	151	21	17-30	0.5			
		G	192	150	24	19-34	0.9			
TIRAP	*rs8177374*	CC	164	128	23	16-30	0.2			
		CT	70	54	34	23-56				
		TT	13	11	8	2-NA				
		C	234	182	24	19-34	0.2			
		T	83	65	28	18-52	0.4			
PGLYR4-24	*rs2916205*	CC	2	0	NA	NA-NA	0.3			
		CT	73	59	18	12-30				
		TT	172	134	26	20-36				
		C	75	59	18	14-34	0.6			
		T	245	193	24	18-30	0.2			
CDKAL1	***rs6908425***	TT	9	6	98	47-NA	0.2			
		CT	66	50	24	16-37				
		CC	172	137	24	16-32				
		T	75	56	28	20-40	0.4	1		
		C	238	187	24	18-30	**0.09**	1.991	0.88-4.49	**0.097**
CD84	*rs6427528*	AA	5	4	18	10-NA	0.8			
		AG	65	53	20	13-37				
		GG	177	136	27	19-36				
		A	70	57	18	13-32	0.5			
		G	242	189	24	19-31	0.7			
IL17RA	*rs4819554*	GG	17	12	34	27-NA	0.3			
		AG	75	59	28	14-46				
		AA	154	122	21	15-30				
		G	92	71	30	20-46	0.2			
		A	229	181	23	17-30	0.2			
IL23R	*rs11209026*	GG	215	164	24	18-31	0.6			
		AG	30	28	22	11-46				
		AA	1	1	110	NA-NA				
		G	245	192	24	18-30	0.6			
		A	31	29	25	11-54	0.5			
TLR2	*rs4696480*	TT	49	39	23	15-37	0.9			
		AT	98	73	24	16-36				
		AA	67	53	18	12-50				
		T	147	112	24	18-34	0.7			
		A	165	126	23	15-31	0.7			
	rs11938228	CC	97	76	21	13-28	0.7			
		AC	86	68	28	16-41				
		AA	42	29	32	18-61				
		C	183	144	24	15-30	0.4			

		A	128	97	30	18-38	0.5			
TLR5	*rs5744174*	AA	86	68	21	14-44	0.9			
		AG	120	90	24	17-36				
		GG	41	35	28	12-54				
		A	206	158	24	18-34	0.8			
		G	161	125	26	19-34	0.9			
TLR9	*rs352139*	TT	54	45	24	16-37	0.6			
		CT	131	103	24	16-36				
		CC	61	44	24	14-54				
		T	185	148	24	18-32	0.3			
		C	192	147	24	17-32	0.8			
PDE3A-SLCO1C1	*rs11045392-rs3794271*	TT	39	34	20	15-46	0.6			
		CT	86	67	23	11-34				
		CC	112	82	27	19-44				
		T	125	101	20	14-31	0.3			
		C	198	149	24	17-34	0.5			
FCGR2A	*rs1801274*	AA	87	69	19	12-31	0.5			
		AG	104	77	28	18-44				
		GG	42	34	27	11-52				
		A	191	146	24	18-34	0.6			
		G	146	111	28	19-37	0.5			
FCGR3A	*rs396991*	AA	84	65	30	19-40	0.8			
		AC	128	99	19	13-31				
		CC	34	28	27	23-56				
		A	212	164	24	18-32	0.8			
		C	162	127	23	16-30	0.5			

MST: median survival time (months); HR: hazard ratio; IC95%: 95% confidence interval; NA: not achieved; Anti-TNF: Tumour Necrosis Factor inhibitor (adalimumab, certolizumab pegol, etanercept and infliximab); PS: Psoriasis.
Statistically significant values are colored in grey, with a tendency to significance in bold.

OPCION 2: SNP RIESGO- SNP ☹ RESPUESTA

Gene	SNPs	Genotype	Drug Survival (months)- ANTI-TNF (N= 247)					Univariate Cox Model		
			N	Events	MST (mo)	IC95%	Log-Rank p-value	HR	IC95%	p-value
HLA-B/ MICA	rs13437088	AA	40	30	34	24-55	0.7			
		AC	108	82	23	16-31				
		CC	98	80	20	12-35				
		A	148	112	25	19-34	0.4			
		C	206	162	23	16-30	0.7			
HLA-C	rs12191877	CC	107	90	14	11-24	0.003	1.87	0.94-3.72	0.677
		CT	124	91	31	23-40		1.157	0.58-2.29	**0.07**
		TT	13	9	35	16-NA		1		
		C	231	181	24	18-30	0.3			
		T	137	100	31	24-40	0.0007	0.609	0.46-0.81	0.00078
TNF	(*TNF-238)* rs361525	GG	204	169	21	16-29	0.009	0.815	0.33-1.99	0.6530
		AG	37	19	44	28-NA		0.401	0.15-1.08	**0.0702**
		AA	6	5	15	7-NA		1		
		G	241	188	24	19-31	0.5			
		A	43	24	37	24-NA	0.007	0.563	0.36-0.86	0.00871
	(*TNF-857)* rs1799724	CC	185	146	24	18-30	0.9			
		CT	60	45	28	16-52				
		TT	2	2	62	37-NA				
		C	245	191	24	18-30	0.7			
		T	62	47	28	16-52	0.8			
	(TNF-*308)* rs1800629	GG	184	137	26	18-37	0.04	1		
		AG	63	56	23	13-30		0.724	0.53-0.99	0.0434
		A	63	56	23	13-80	0.04	0.724	0.53-0.99	0.0434
	(TNF-1031) rs1799964	TT	145	124	20	15-27	0.003	0.947	0.55-1.62	0.8453
		CT	85	54	44	25-70		0.552	0.31-0.98	0.0438
		CC	17	15	20	11-NA		1		
		T	230	178	24	18-31	0.4			
		C	102	69	36	23-56	0.004	0.648	0.48- 0.87	0.00442
TNFRSF1B	rs1061622	TT	153	127	24	15-31	0.2			
		GT	70	48	29	23-54				
		GG	24	18	17	11-56				
		T	223	175	24	19-31	0.9			
		G	94	66	28	19-44	0.1			
TNFAIP3	rs610604	GG	24	18	15	8-NA	0.9			
		GT	124	96	24	17-37				
		TT	98	78	25	16-36				
		G	148	114	23	17-34	0.8			
		T	222	174	24	19-31	0.8			
IL1B	*rs1143623*	CC	146	114	23	17-31	0.6			
		CG	81	62	30	16-38				
		GG	20	17	15	8-46				
		C	227	176	24	19-31	0.4			
		G	101	79	28	14-37	0.6			
	rs1143627	GG	28	24	21.5	8-44	0.4			
		AG	105	77	34	20-41				
		AA	114	92	20	16-28				
		G	133	101	30	19-37	0.5			
		A	219	169	24	18-34	0.4			
IL6	*rs1800795*	CC	21	14	17	11-NA	0.7			

		CG	101	75	27	18-37				
		GG	125	104	23	16-34				
		C	122	89	25	18-37	0.4			
		G	226	179	24	19-34	0.8			
IL12β	*rs3213094*	CC	162	126	25	19-36	0.4			
		CT	78	60	24	13-37				
		TT	6	6	17.5	3-NA				
		C	240	186	24	19-31	0.2			
		T	84	66	23	13-32	0.5			
	rs2546890	AA	55	43	18	12-40	0.8			
		AG	138	108	23	17-34				
		GG	54	42	30	23-52				
		A	193	151	21	17-30	0.5			
		G	192	150	24	19-34	0.9			
TIRAP	*rs8177374*	CC	164	128	23	16-30	0.2			
		CT	70	54	34	23-56				
		TT	13	11	8	2-NA				
		C	234	182	24	19-34	0.2			
		T	83	65	28	18-52	0.4			
PGLYR4-24	*rs2916205*	CC	2	0	NA	NA-NA	0.3			
		CT	73	59	18	12-30				
		TT	172	134	26	20-36				
		C	75	59	18	14-34	0.6			
		T	245	193	24	18-30	0.2			
CDKAL1	***rs6908425***	TT	9	6	98	47-NA	0.2			
		CT	66	50	24	16-37				
		CC	172	137	24	16-32				
		T	75	56	28	20-40	0.4	1		
		C	238	187	24	18-30	**0.09**	1.991	0.88-4.49	**0.097**
CD84	*rs6427528*	AA	5	4	18	10-NA	0.8			
		AG	65	53	20	13-37				
		GG	177	136	27	19-36				
		A	70	57	18	13-32	0.5			
		G	242	189	24	19-31	0.7			
IL17RA	*rs4819554*	GG	17	12	34	27-NA	0.3			
		AG	75	59	28	14-46				
		AA	154	122	21	15-30				
		G	92	71	30	20-46	0.2			
		A	229	181	23	17-30	0.2			
IL23R	*rs11209026*	GG	215	164	24	18-31	0.6			
		AG	30	28	22	11-46				
		AA	1	1	110	NA-NA				
		G	245	192	24	18-30	0.6			
		A	31	29	25	11-54	0.5			
TLR2	*rs4696480*	TT	49	39	23	15-37	0.9			
		AT	98	73	24	16-36				
		AA	67	53	18	12-50				
		T	147	112	24	18-34	0.7			
		A	165	126	23	15-31	0.7			
	rs11938228	CC	97	76	21	13-28	0.7			
		AC	86	68	28	16-41				
		AA	42	29	32	18-61				
		C	183	144	24	15-30	0.4			
		A	128	97	30	18-38	0.5			

TLR5	*rs5744174*	AA	86	68	21	14-44	0.9			
		AG	120	90	24	17-36				
		GG	41	35	28	12-54				
		A	206	158	24	18-34	0.8			
		G	161	125	26	19-34	0.9			
TLR9	*rs352139*	TT	54	45	24	16-37	0.6			
		CT	131	103	24	16-36				
		CC	61	44	24	14-54				
		T	185	148	24	18-32	0.3			
		C	192	147	24	17-32	0.8			
PDE3A-SLCO1C1	*rs11045392-rs3794271*	TT	39	34	20	15-46	0.6			
		CT	86	67	23	11-34				
		CC	112	82	27	19-44				
		T	125	101	20	14-31	0.3			
		C	198	149	24	17-34	0.5			
FCGR2A	*rs1801274*	AA	87	69	19	12-31	0.5			
		AG	104	77	28	18-44				
		GG	42	34	27	11-52				
		A	191	146	24	18-34	0.6			
		G	146	111	28	19-37	0.5			
FCGR3A	*rs396991*	AA	84	65	30	19-40	0.8			
		AC	128	99	19	13-31				
		CC	34	28	27	23-56				
		A	212	164	24	18-32	0.8			
		C	162	127	23	16-30	0.5			

MST: median survival time (months); HR: hazard ratio; IC95%: 95% confidence interval; NA: not achieved; Anti-TNF: Tumour Necrosis Factor inhibitor (adalimumab, certolizumab pegol, etanercept and infliximab); PS: Psoriasis.
Statistically significant values are colored in grey, with a tendency to significance in bold.
